# Strategies to Improve the Antitumor Effect of Immunotherapy for Hepatocellular Carcinoma

**DOI:** 10.3389/fimmu.2021.783236

**Published:** 2021-11-26

**Authors:** Rui Xing, Jinping Gao, Qi Cui, Qian Wang

**Affiliations:** ^1^ Department of Oncology, Shengjing Hospital of China Medical University, Shenyang, China; ^2^ Department of Oncology, North War Zone General Hospital, Shenyang, China; ^3^ Department of Cold Environmental Medicine, College of High Altitude Military Medicine, Third Military Medical University (Army Medical University), Chongqing, China

**Keywords:** hepatocellular carcinoma, immune checkpoint inhibitors, combination therapy, antiangiogenic drugs, locoregional therapies

## Abstract

Hepatocellular carcinoma (HCC), one of the most fatal malignancies in the world, is usually diagnosed in advanced stages due to late symptom manifestation with very limited therapeutic options, which leads to ineffective intervention and dismal prognosis. For a decade, tyrosine kinase inhibitors (TKIs) have offered an overall survival (OS) benefit when used in a first-line (sorafenib and lenvatinib) and second-line setting (regorafenib and cabozantinib) in advanced HCC, while long-term response remains unsatisfactory due to the onset of primary or acquired resistance. Recently, immunotherapy has emerged as a promising therapy in the treatment of several solid tumors, such as melanoma and non-small cell lung cancer. Moreover, as the occurrence of HCC is associated with immune tolerance and immunosurveillance escape, there is a potent rationale for employing immunotherapy in HCC. However, immunotherapy monotherapy, mainly including immune checkpoint inhibitors (ICIs) that target checkpoints programmed death-1 (PD-1), programmed death-ligand 1 (PD-L1), and the cytotoxic T lymphocyte antigen-4 (CTLA-4), has a relatively low response rate. Thus, the multi-ICIs or the combination of immunotherapy with other therapies, like antiangiogenic drugs and locoregional therapies, has become a novel strategy to treat HCC. Combining different ICIs may have a synergistical effect attributed to the complementary effects of the two immune checkpoint pathways (CTLA-4 and PD-1/PD-L1 pathways). The incorporation of antiangiogenic drugs in ICIs can enhance antitumor immune responses *via* synergistically regulating the vasculature and the immune microenvironment of tumor. In addition, locoregional treatments can improve antitumor immunity by releasing the neoplasm antigens from killed tumor cells; in turn, this antitumor immune response can be intensified by immunotherapy. Therefore, the combination of locoregional treatments and immunotherapy may achieve greater efficacy through further synergistic effects for advanced HCC. This review aims to summarize the currently reported results and ongoing trials of the ICIs-based combination therapies for HCC to explore the rational combination strategies and further improve the survival of patients with HCC.

## Introduction

Hepatocellular carcinoma (HCC), the third leading cause of cancer-related deaths in the world, has been referred to as a highly devastating malignancy, with a low 5-year survival rate of about 18% (1, 2). In the early stages, patients with HCC could receive therapies with curative intent such as resection, radiofrequency or microwave ablation, and liver transplant, while most patients are diagnosed with advanced stage cancer due to late symptom manifestation. Even after curative therapy, the majority of patients will develop recurrence or metastases within 5 years and the systemic therapy still serves as the mainstay in the treatment for HCC (3, 4). For many years, the commonly used therapeutic regimens for advanced HCC in clinical practice are mainly targeted therapy based on antiangiogenic drugs, radiotherapy, and chemotherapy. However, these treatments are characterized by low response rates, high recurrence rate, and limited improvements in overall survival (OS) (5). Hence, it is urgent to find an optimal treatment strategy to improve the effect of the treatment for HCC.

In recent years, application of immune checkpoint inhibitors (ICIs), especially programmed cell death protein 1 (PD-1), programmed cell death ligand 1 (PD-L1), and cytotoxic T lymphocyte antigen 4 (CTLA-4) monoclonal antibodies against immune regulatory checkpoints on immune and tumor cells, represents a major breakthrough in the treatment of many solid tumors, such as HCC (6, 7). At present, since pembrolizumab and nivolumab, anti-PD-1 humanized antibodies, have shown encouraging signs of efficacy in relapsed and refractory HCC patients who previously received sorafenib in several trials, these two drugs were approved by the US Food and Drug Administration (FDA) as a second-line treatment for these patients (8, 9). However, the objective response rate (ORR) is relatively low, with 20% and 16.9% ORR for nivolumab and pembrolizumab, respectively (8, 9). Therefore, the large percentage of patients who fail to respond to immune monotherapy drives the exploration of therapeutic strategies to improve the effect of immunotherapy against HCC.

Despite the setbacks, many studies have shown that the combination of ICIs with other treatment approaches such as tyrosine kinase inhibitors (TKI) or local therapies, and even multi-ICI combinatorial therapies, has presented an obvious effect in the population treated for HCC (10). One of the most successful examples is that the combination of atezolizumab (PD-L1 inhibitor) and bevacizumab has been approved by US FDA as a first-line treatment for patients with unresectable HCC based on unprecedented results of the IMbrave150 trial (11–13). This review focuses on the currently reported results and ongoing trials of combination-based ICIs in HCC so as to optimize treatment strategies to overcome resistance to ICIs and acquire satisfactory effects in the treatment of HCC.

## Mechanism of HCC Immune Tolerance

The majority of HCC arises from the underlying chronic inflammation including chronic hepatitis B virus (HBV) and hepatitis C virus (HCV) infections, alcoholic steatohepatitis, non-alcoholic steatohepatitis, and exposure to toxic agents such as aflatoxin (14). These chronic inflammations result in the impairment of immune surveillance and dysregulation of immune environment by damaging the reticuloendothelial system (RES) of the liver, thus escaping the immune surveillance of the host and creating an immunosuppressive surrounding (15). In addition, for HCC, a typical inflammation-associated malignancy, immune evasion can boost the tumor immunogenicity and induce DNA damage and genetic aberrations, which plays a vital role in the initiation, evolution, and progression of neoplasms (16, 17).

**Figure 1:**
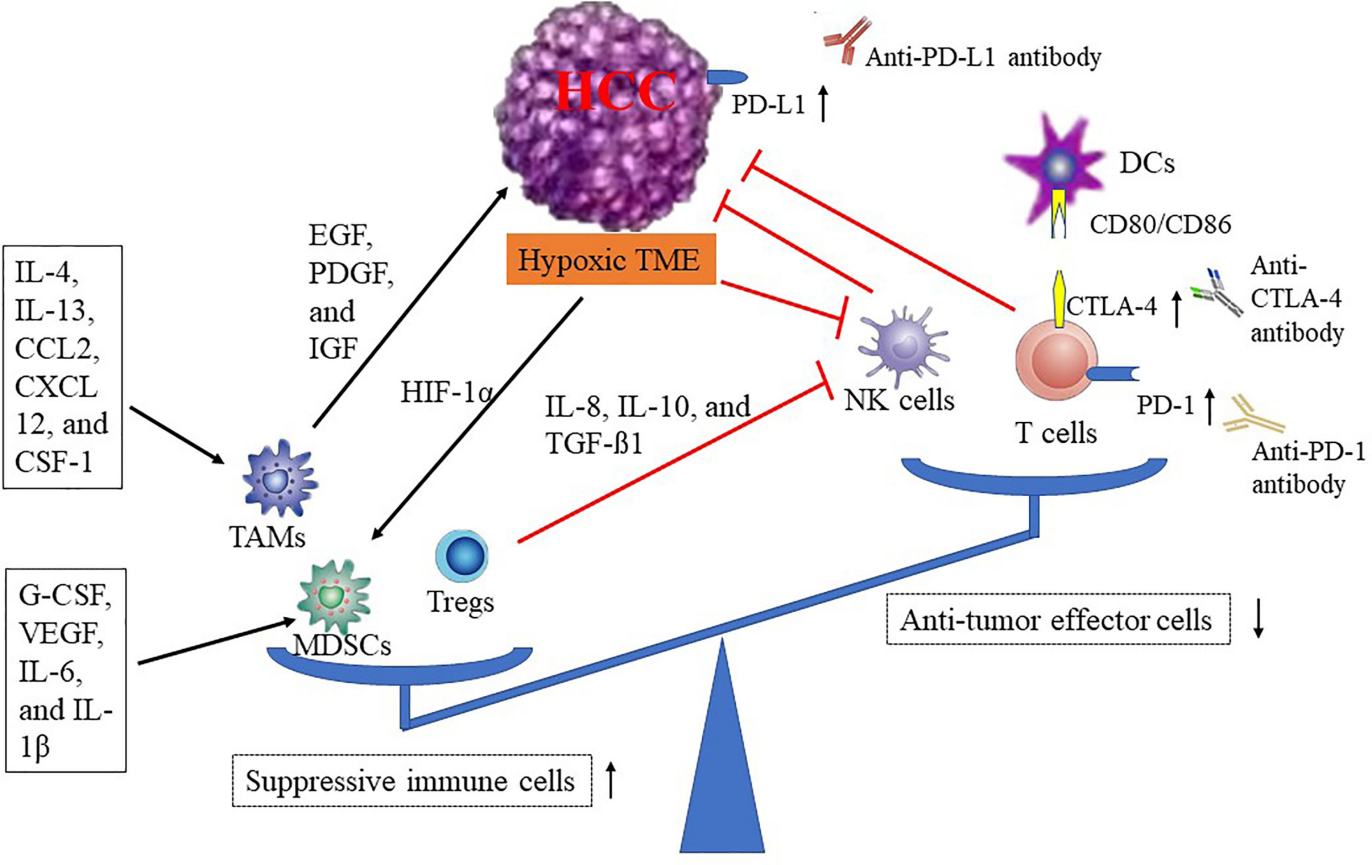
Mechanisms of HCC immune tolerance. CCL-2, C-C motif chemokine ligand 2; CTLA-4, cytotoxic T-lymphocyte antigen-4; CSF-1, colony-stimulating factor; DCs, dendritic cells; CXCL-12, C-X-C motif chemokine ligand 12; EGF, epidermal growth factor; HCC, hepatocellular carcinoma; HIF-1α, hypoxia-inducible factor 1α; IGF, insulin-like growth factor; IL, interleukin; NK cells, natural killer cells; MDSCs, myeloid-derived suppressor cells; PD-1, programmed death-1; PD-L1, programmed death-ligand 1; PDGF, platelet-derived growth factor; TMAs, tumor-associated macrophage; TME, tumor microenvironment; Tregs, regulatory T cell; VEGF, vascular endothelial growth factor.

The tumor microenvironment (TME) of HCC is characterized by immune suppression *via* a variety of mechanisms, including the recruitment of suppressive immune cells [tumor-associated macrophages (TAMs), myeloid-derived suppressor cells (MDSCs), and regulatory T cells (Tregs)], the reduction of antitumor effector cells [natural killer cells (NK cells) and dendritic cells (DCs)], the change of cytokine level, and the increase of immune checkpoint proteins (PD-1/PD-L1 and CTLA-4), and these mechanisms are interactive (18) (**Figure 1**). As the largest number of infiltrating inflammatory cells in the TME, TAMs can suppress antitumor immune effects in HCC (19, 20). Some studies have revealed that several immunosuppressive cytokines, such as interleukin-4 (IL-4), interleukin-13 (IL-13), C-C motif chemokine ligand 2 (CCL-2), C-X-C motif chemokine ligand 12 (CXCL12), and colony-stimulating factor (CSF-1), can promote TAMs differentiation, resulting in reduction of innate or adaptive immunity (21, 22). In turn, the TAMs can block antitumor immune responses and accelerate tumor progression by increasing the expression of cytokines and chemokines, such as platelet-derived growth factor (PDGF), epidermal growth factor (EGF), and insulin-like growth factor (IGF) (23). Hypoxia, a typical characteristic of the TME, can induce the recruitment of MDSCs (the powerful immunosuppressive cells) in the TME through hypoxia-inducible factor 1α (HIF-1α) (24) (**Figure 1**). In addition, a variety of tumor-originated cytokines, such as G-CSF, VEGF, IL-6, and IL-1β, have been proven to promote MDSCs accumulation (25–27). Emerging evidence indicates that increased populations of Tregs in peripheral blood of patients are associated with invasiveness of HCC and inhibition of effective antitumor responses in HCC (28, 29). Furthermore, NK cells, the pivotal component of the innate immune system, are affected by the hypoxic stress in HCC tissue, leading to dysfunction (30). There are studies that show that the infiltrated MDSCs and TAMs can damage effector T cells, decrease NK cells cytotoxicity, reduce tumor-infiltrating lymphocytes (TILs), and expand immune checkpoint signaling in HCC (31–33). In addition, Tregs can impair the function of NK cells *via* the release of several cytokines (IL-8, IL-10, and TGF-β1) (34). With respect to immune checkpoints associated with tumor cell immune evasion, such as CTLA-4, PD-1, PD-L1, T-cell immunoglobulin and ITIM domain (TIGIT) (35), lymphocyte activation gene 3 (LAG-3) (36), and T-cell immunoglobulin-3 (TIM-3) (36), these inhibitory receptors/ligands inhibit the antitumor immune response by altering their expression levels (37) (**Figure 1**). Therefore, there are strong reasons to treat HCC patients with immunotherapies.

## Rationality of Immunotherapy

Immune checkpoints, mainly including PD-1/PD-L1 and CTLA-4, play a key role in the initiation and preservation of tumor immune escape. The first pathway is PD-1/PD-L1. PD-1, expressed on activated B cells, T cells, DCs, and NK cells, can produce inhibitory signals by binding with PD-L1, resulting in inhibition of activation of these immune cells, and protecting the tumor cell from attack (38). Another one is CTLA-4, an inhibitory immune checkpoint, expressed on activated T cells. It is known that the activation of naive T cells is regulated by the co-stimulatory [the interaction between CD28 on T cell and CD80/86 on antigen presenting cell (APC)] and co-inhibitory (CTLA-4 on T cell binding CD80/86 on APC) function of immune checkpoint receptors (39, 40). That is, the binding of CD28 to B7-1/2 activates T cell, while the binding of CTLA-4 to CD80/86 is a negative signal and in turn inhibits T-cell proliferation. Therefore, anti-CTLA-4 antibodies can enhance T cells’ antitumor effect by blocking the binding of CTLA-4 on T cell to CD80/86 on APC. In addition, CTLA-4 is also expressed on Tregs (a suppressive immune cell), which plays an important role in regulating Tregs function. As a co-inhibitory receptor, CTLA-4 on Tregs inhibits co-stimulatory signaling through higher affinity binding to CD80/86 on APC compared with the co-stimulatory receptor CD28 on T cells, resulting in the impairment of the antitumor immune response and acceleration of immune evasion of tumor cells (41). Hence, CTLA-4 blockage may reinforce antitumor responses by attenuating Tregs function. Furthermore, a study demonstrated that anti-CTLA-4 drugs can efficiently deplete Tregs through Fc-mediated antibody-dependent cellular cytotoxicity, leading to increased antitumor immune responses (42). Therefore, ICIs, including anti-PD-1 (pembrolizumab and nivolumab), anti-PD-L1 (durvalumab and atezolizumab), and anti-CTLA-4 (tremelimumab and ipilimumab), are promising agents that can facilitate the proliferation of immune cells and reinforce antitumor immune response by blocking the ligand binding site or inhibiting the expression of PD-1 and CTLA-4 in the treatment of patients with HCC. Interestingly, in patients with HCC, immune checkpoint-related molecules (PD-1, PD-L1, and CTLA-4) are usually overexpressed due to long-term chronic inflammation, resulting in the apoptosis of CD8^+^ T cells and a decreased activity of these cells against tumor (43, 44). Thus, the aforementioned can provide a potent rationale for the utilization of ICIs as treatment of patients with HCC.

*Nivolumab*, an anti-PD-1 antibody, can inhibit the interaction with PD-L1 on tumor cells by binding to PD-1 receptor on T cells, leading to restoration of the antitumor activity of T cells. An open-label, non-comparative, phase I/II study (CheckMate 040, NCT01658878, **Table 1**) was performed to evaluate the safety and efficacy of nivolumab in patients with advanced HCC (9). This study enrolled 262 patients with or without previous therapy of sorafenib. In the dose escalation phase (*n* = 48), nivolumab has a favorable safety profile, and the grade 3/4 treatment-related adverse events (TRAEs) occurred in 12 of 48 (25%) patients. TRAEs that occurred in more than 10% of patients mainly included rash (11 patients), aspartate aminotransferase (AST) increase (10 patients), lipase increase (10 patients), amylase increase (9 patients), pruritus (9 patients), and alanine aminotransferase (ALT) increase (7 patients). Based on the results from the dose-escalation phase, a dosage of 3 mg/kg was selected for the dose-expansion phase. In this phase (*n* = 214), this regimen achieved an ORR of 20% (95% CI, 15–26), including three complete responses (CR) and 39 partial responses (PR). Stable disease (SD) was observed in 45% (96/214) of patients, and thus disease control rate (DCR) reaches 64% (138/214) in patients, according to the Solid Tumors (RECIST) 1.1 criteria. The 6-month progression-free survival (PFS) and the 9-month PFS rate were 37% (95% CI: 30–43) and 28% (95% CI: 22–35), respectively. The median time to progression (MTP) was 4.1 months (95% CI: 3.7–5.5). The 6-month OS was 83% (95% CI: 78–88), and the 9-month OS was 74% (95% CI: 67–79). Interestingly, further stratified analysis by baseline tumor cell PD-L1 status has shown that patients with PD-L1 expression on tumor cell of at least 1% have higher ORR compared with patients with PD-L1 expression on tumor cell of less than 1% (26% *vs*. 19%). Additionally, in the Asian cohort subanalysis of CheckMate 040, patients with HBV, HCV, or those without viral hepatitis had ORR of 13%, 14%, and 21%, respectively (45). Therefore, the PD-1/PD-L1 expression and etiology may become the potentially valuable biomarkers to guide the use of nivolumab in HCC, which needs further clinical trials.

**Table 1:** Clinical trials investigating the efficacy of ICIs alone or multi-ICIs treatment in HCC.

Study design	Phase	Patients (*n*)	Status (endpoints or results)	ClinicalTrials.gov Identifier
Open-label, non-randomized, parallel assignment: the effectiveness, safety, and tolerability of nivolumab or nivolumab in combination with other agents (sorafenib, ipilimumab, or cabozantinib)	I/II	Advanced HCC (659)	Active, not recruiting (ORR: 20% (95% CI 15–26); DCR: 64%; 6-month PFS rate: 37% (95% CI 30–43); 9-month PFS rate: 28% (95% CI 22–35); the median TTP: 4.1 months (95% CI 3.7–5.5); 6-month OS: 83% (95% CI 78–88); 9-month OS: 74% (95% CI 67–79)	NCT01658878
Open-label, randomized, parallel assignment: nivolumab *vs*. sorafenib as a first treatment	III	Advanced HCC (743)	Active, not recruiting (the median OS: 16.4 *vs*. 14.7 months for nivolumab *vs*. sorafenib (HR 0.85 [95% CI: 0.72–1.02]; *p* = 0.0752); ORR: 15% for nivolumab and 7% for sorafenib)	NCT02576509
Open-label, non-randomized, parallel assignment: pembrolizumab as monotherapy	II	Advanced HCC (156)	Active, not recruiting (ORR: 17% (95% CI 11–26); TRAEs: 73%)	NCT02702414
Randomized, placebo-controlled, parallel assignment: pembrolizumab *vs*. BSC as second-line therapy	III	Advanced HCC with prior systemic therapy (413)	Active, not recruiting (PFS: 3.0 *vs*. 2.8 months for placebo *vs*. pembrolizumab [HR: 0.78; one sided *p* = 0.0209); OS: 13.9 *vs*. 10.6 months for placebo *vs*. pembrolizumab (HR: 0.78; one sided *p* = 0.0238)]	NCT02702401
Participant, investigator, randomized, parallel assignment: pembrolizumab + BSC *vs*. placebo + BSC as second-line therapy	III	Asian subjects with previously systemically treated advanced HCC (450)	Active, not recruiting (primary endpoints: OS secondary endpoints: PFS; ORR; DOR; DCR; TTP; AEs)	NCT03062358
Randomized, double-blinded, two-arm, sequential assignment: pembrolizumab *vs*. placebo as adjuvant therapy in participants with HCC and complete radiological response after surgical resection or local ablation	III	HCC (950)	Recruiting (primary endpoints: RFS; OS secondary endpoints: AEs; quality of life)	NCT03867084
Open-label, multicenter, single group assignment: the safety, tolerability, and pharmacokinetics of durvalumab	I/II	HCC (40)	Completed [ORR: 10.3% (95% CI 2.9%–24.2%); DCR: 33.3% (95% CI 19.1%–50.2%); median OS: 13.2 months (95% CI 6.3%–21.1%); TRAEs: 80.0%; Grade 3/4 TRAEs: 20.0%]	NCT01693562
Open-label, non-randomized, single group assignment: tremelimumab	II	Advanced HCC (20)	Completed (PR rate: 17.6%; DCR: 76.4%; median PFS: 6.48 months)	NCT01008358
Open-label, randomized, parallel assignment: durvalumab+tremelimumab	II	Advanced HCC (433)	Active, not recruiting (ORR: 15%, 16-week DCR: 57%; ≥3 TRAEs: 20%)	NCT02519348
Open-label, randomized, parallel assignment: durvalumab+tremelimumab *vs*. sorafenib as first-line treatment	III	Unresectable HCC with no prior systemic therapy (1504)	Recruiting (primary endpoints: OS secondary endpoints: TTP; PFS; ORR; DCR; DOR)	NCT03298451
Open-label, randomized, parallel assignment: nivolumab+ipilimumab *vs*. sorafenib/lenvatinib as first-line treatment	III	Advanced HCC (662)	Recruiting (primary endpoints: OS secondary endpoints: ORR; DOR; TTSD)	NCT04039607

AEs, adverse events; BSC, best supportive care; CI, confidence interval; DCR, disease control rate; DOR, duration of response; HCC, hepatocellular carcinoma; HR, hazard ratio; ICIs, immune checkpoint inhibitors; ORR, objective response rate; OS, overall survival; PFS, progression-free survival; PR, partial remission; RFS, recurrence-free survival; TRAEs, treatment-related adverse events; TTP, time to tumor progression; TTR, time to recurrence; TTSD, time to symptom deterioration.

Due to the lack of randomized control arms for CheckMate 040, a subsequent CheckMate 459 (NCT02576509, **Table 1**), the phase III randomized controlled trial, further tests the therapeutic potential of nivolumab in patients with advanced HCC by comparing nivolumab with sorafenib (46). In this trial, 743 patients were enrolled and treated randomly with nivolumab (*n* = 371) and sorafenib (*n* = 372), with a minimum follow-up of 22.8 months at data cutoff. The median OS was 16.4 months for nivolumab compared to 14.7 months for sorafenib (HR 0.85 [95% CI: 0.72–1.02]; *p* = 0.0752). ORR was 15% for nivolumab (14 patients with CR) *vs*. 7% for sorafenib (5 patients with CR). Of note, ORRs were 28% (20/71) in patients with baseline tumor PD-L1 expression ≥1% and 12% (36/295) in patients with baseline tumor PD-L1 expression<1%. Grade 3/4 TRAEs were less in the nivolumab arm [22% (81 patients)] compared with the sorafenib arm [49% (179 patients)]. Nivolumab showed a manageable safety profile consistent with previous studies. Albeit not statistically significant for OS, the primary endpoint, these results suggest a clinical benefit of nivolumab for advanced HCC, with meaningful improvements in response rate and survival, especially for the patients who have PD-L1 expression ≥1%. Therefore, nivolumab was granted accelerated approval by FDA as a second-line treatment of patients with advanced HCC after sorafenib (47).

*Pembrolizumab* is another anti-PD-1 antibody. A non-randomized, open-label phase II trial (KEYNOTE-224), was conducted to assess the efficacy and safety of pembrolizumab in patients with HCC following progression on sorafenib (48). In this study, a total of 104 patients were treated with an objective response in 18 of 104 patients (ORR: 17%; 95% CI: 11–26), including 1 (1%) CR and 17 (16%) PR. Forty-six (44%) patients had SD. Seventy-six of 104 (73%) patients had TRAEs, such as increased AST concentration (7%), increased ALT concentration (4%), and fatigue (4%), which were serious in 16 (15%) patients. Additionally, this study assessed the correlations between PD-L1 expression and clinical outcomes. There are two evaluation methods for PD-L1 expression, including combined positive score (CPS), described as the number of PD-L1-positive cells (tumor cells, macrophages, and lymphocytes) divided by the total number of tumor cells and multiplied by 100, and the tumor proportion score (TPS), described as the percentage of viable tumor cells with partial or complete membrane staining of PD-L1 (≥1%) to all viable tumor cells in the sample. Of the 52 participants with data available for PD-L1 expression, there were 22 (42%) patients with CPS ≥ 1 and 7 (13%) patients with TPS ≥ 1%. ORR was 32% (7 of 22 patients) for patients with CPS ≥ 1 *vs*. 20% (6 of 30 patients) for patients with CPS<1 (*p* = 0.021), and 43% (3 of 7 patients) for TPS ≥ 1% *vs*. 22% (10 of 45 patients) for TPS <1% (*p* = 0.088). PD-L1 expression in tumor and immune cells, as assessed by the CPS, could improve the predictive value of PD-L1 as a biomarker because the difference of TPS was not significant. Based on this study, a subsequent randomized, placebo-controlled phase III trial (KEYNOTE-240, NCT 02576509, **Table 1**) was conducted. Although, compared with placebo (*n* = 135), the differences in PFS and OS did not reach the pre-specified statistical significance, pembrolizumab (*n* = 278) improved PFS (3.0 *vs*. 2.8 months; HR: 0.78; one sided *p* = 0.0209) and OS (13.9 *vs*. 10.6 months; HR: 0.78; one sided *p* = 0.0238) with the safety profile consistent with that previously reported in pembrolizumab studies (8). Hence, pembrolizumab got the FDA’s approval as a second-line treatment for advanced HCC treated with prior sorafenib (49). At present, there are two ongoing further phase III trials, namely, the MK-3475-394 study (NCT03062358), which tests the safety and efficacy of pembrolizumab or placebo given with the best supportive care as a second-line therapy in Asian patients with advanced HCC, and the MK-3475-937 trial (NCT03867084), which assesses the efficacy and safety of pembrolizumab *vs*. placebo as an adjuvant therapy in subjects with HCC and complete radiological response after local ablation or surgical resection (**Table 1**).

*Durvalumab*, a fully human anti-PD-L1 mAb, has shown an acceptable safety profile and promising antitumor activity for patients with advanced HCC pre-treated with sorafenib in a multicenter, open-label, single group assignment phase I/II study (NCT01693562, **Table 1**) (50). The ORR was 10.3% (95% CI 2.9–24.2%) and the DCR was 33.3% (95% CI 19.1–50.2%). The median OS is 13.2 months (95% CI 6.3–21.1%). Eighty percent of patients experience TRAEs, most commonly fatigue (27.5%), pruritus (25.0%), and elevated AST (22.5%). Grade 3/4 TRAEs were reported in 20.0% of patients, and there were no deaths due to TRAEs.

*Tremelimumab*, a monoclonal antibody that blocks CTLA-4, was tested for the antitumor and antiviral effect in patients with HCC and chronic infections of HCV in an open label, non-randomized phase II trial (NCT 01008358, **Table 1**) (51). This study had shown a good safety profile, and the partial response rate was 17.6% and DCR was 76.4%, with a median PFS of 6.48 months. In addition, a prominent drop in viral load was observed. These findings suggest that tremelimumab could be particularly promising for patients with advanced HCC developed on HCV-induced liver cirrhosis. David Agdashian et al. found that tremelimumab treatment can lead to an activation of tumor-specific T cells, a decrease in T-cell clonality, and an influx of CD3^+^ CD8^+^ T cells in the tumor, with profound clinical and immunological responses in HCC patients (52).

## Multi-ICIs Combination

As described above, although single-agent immunotherapy has achieved encouraging results in HCC, its response rate remains unsatisfactory. Interestingly, a study demonstrated that the CTLA-4 inhibitor in combination with the PD-1/PD-L1 inhibitor can result in a nonredundant effect (53). CTLA-4 can inhibit the proliferation of T cells mainly in lymph nodes at the initial stage of the immune response, while PD-1 plays a crucial role in peripheral tissues including tumor tissue by suppressing previously activated T cells at the later stages of this response (53). In addition, CTLA-4 blockade can induce a robust proliferative signature predominantly in a subset of transitional memory T cells. In contrast to CTLA-4 blockade, PD-1-regulated genes are not associated with proliferation signature and PD-1 blockade can induce upregulation of several cytolysis and natural killer-associated genes (54). Because of the differences in timing, location, and nonoverlapping effects between the PD-1/PD-L1 and CTLA-4 signaling pathways, combination therapy concurrently targeting these two immune checkpoints may achieve the potential synergistic effects in the treatment of HCC.

Although durvalumab (against PD-L1 immune checkpoint) and tremelimumab (against CTLA-4 immune checkpoint) have demonstrated impressive efficacy in monotherapy as mentioned earlier, its result is not as satisfying as expected. Thus, the combination of durvalumab and tremelimumab is worth exploring. An open-label, randomized phase I/II study (NCT02519348) was conducted to evaluate the safety and effect of these combination in unresectable HCC (55). This trial illustrated an ORR of 15%, a DCR at 16 weeks of 57%, and an acceptable safety profile in patients with unresectable HCC who received durvalumab + tremelimumab treatment. Twenty-four (60%) patients had ≥1 TRAEs. The most common TRAEs were fatigue (20%), pruritus (18%), increased ALT (18%), and increased AST (15%). Twenty percent of patients had ≥3 TRAEs. The most common grade ≥3 TRAEs were an asymptomatic rise in AST (10%). Based on the result from this study, an ongoing expansive study (HIMALAYA study, NCT03298451, **Table 1**) was prompted. This is a multicenter, randomized phase III trial aimed to assess the safety and effect of combination therapy of tremelimumab and durvalumab *vs*. sorafenib as first-line treatment of patients with unresectable HCC (56).

For another combination (ipilimumab plus nivolumab, monoclonal antibodies against CTLA-4 immune checkpoints and PD-1 immune checkpoints, respectively), there was a Checkmate 040 randomized clinical study (57), a multicenter, open-label, multicohort, phase I/II trial, designed to test the efficacy and safety of varying dosages of combination therapy for advanced HCC patients who previously received sorafenib. A total of 148 sorafenib-treated patients were randomized 1:1:1 into three dosing arms, including arm A (*n* = 50): nivolumab 1 mg/kg + ipilimumab 3 mg/kg every 3 weeks (four doses) followed by nivolumab 240 mg every 2 weeks, arm B (*n* = 49): nivolumab 3 mg/kg + ipilimumab 1 mg/kg every 3 weeks (four doses) followed by nivolumab 240 mg every 2 weeks, and arm C (*n* = 49): nivolumab 3 mg/kg every 2 weeks + ipilimumab 1 mg/kg every 6 weeks, until discontinuation due to disease progression or intolerable toxicity. The median follow-up was 30.7 months. In arms A, B, and C, investigator-assessed ORR was 32% (95% CI, 20%–47%), 27% (95% CI, 15%–41%), and 29% (95% CI, 17%–43%), respectively. Encouragingly, patients in arm A had the most promising OS compared to those in arms B and C: 23.0 months (95% CI, 9.4–not reached) *vs*. 12.5 months (95% CI, 7.6–16.4) *vs*. 12.7 months (95% CI, 7.4–33.0), respectively.

All grade TRAEs were seen in 46 of 49 patients (94%) in arm A, 35 of 49 patients (71%) in arm B, and 38 of 48 patients (79%) in arm C. Although this study revealed higher rates of TRAEs for nivolumab + ipilimumab regimens than nivolumab monotherapy (57), the types of events were similar to nivolumab or ipilimumab monotherapy, without new safety signals. Based on the promising results from this study, nivolumab 1 mg/kg plus ipilimumab 3 mg/kg every 3 weeks for four doses and then maintenance of nivolumab (240 mg every 2 weeks) has received accelerated approval as a second-line treatment for HCC in the United States. However, this study has several limitations, such as small patient population, lack of a comparator arm, and lack of patient stratification. Therefore, the larger, randomized, active comparator-controlled clinical trials are required to explore the clinical benefits of nivolumab plus ipilimumab regimens. At present, the CheckMate 9DW trial (NCT04039607, **Table 1**), a randomized, multicenter, phase III study, is ongoing to compare the OS of ipilimumab plus nivolumab *vs*. sorafenib or lenvatinib in participants with advanced HCC who have not received prior systemic therapy.

## Combining With Antiangiogenic Drugs

In the past decades, molecular-targeted therapies have made a significant breakthrough for advanced HCC. Sorafenib, a multiple TKI, as the only first-line systemic treatment of patients with advanced HCC for a long time, exerts antitumor effects by suppressing vascular endothelial growth factor receptor (VEGFR), platelet-derived growth factor receptor (PDGFR), Raf-1, and B-Raf (58). Another TKI (lenvatinib) can decrease tumor vascular permeability and inhibit tumor neovessel maturation and assembly through targeting VEGFR1–3, fibroblast growth factor receptor (FGFR)1–4, PDGFR-α, c-Kit protein, and RET protein. In a randomized phase III trial (REFLECT trial), Masatoshi Kudo et al. demonstrated that lenvatinib was non-inferior to sorafenib in OS for untreated advanced HCC. Based on this result, lenvatinib was approved for the first-line therapy in advanced HCC (59). In recent years, several targeted drugs, including regorafenib, cabozantinib, sunitinib, linifanib, brivanib, tivantinib, and ramucirumab, have received approval as a second-line treatment for HCC patients after first-line sorafenib or lenvatinib disease progression (60). However, for the majority of patients with HCC, monotherapy with targeted therapies demonstrated limited clinical benefits, such as a survival rate of only 3 months offered in the treatment of sorafenib and lenvatinib for patients with inoperable HCC (59, 61).

**Figure 2:**
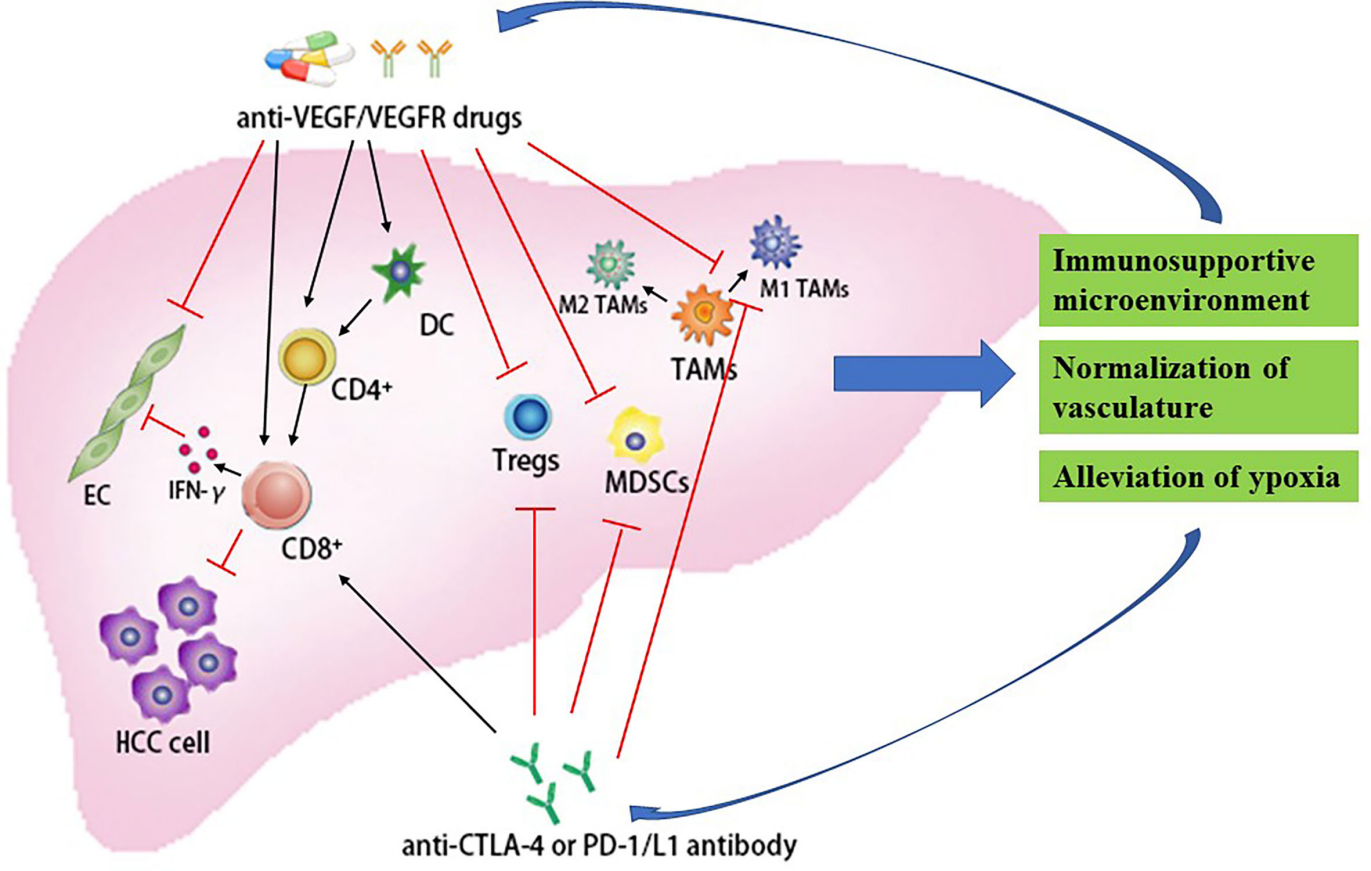
Synergistic effect of immune checkpoint blockade in combination with antiangiogenic drugs for the treatment of HCC. CD4^+^, CD4-positive T-lymphocytes; CD8^+^, CD8-positive T-lymphocytes; CTLA-4, cytotoxic T-lymphocyte antigen-4; DC, dendritic cells; EC, endothelial cells; HCC, hepatocellular carcinoma; IFN-γ, interferon-γ; MDSCs, myeloid-derived suppressor cells; PD-1, programmed death-1; PD-L1, programmed death-ligand 1; TMAs, tumor-associated macrophage; Tregs, regulatory T cell; VEGF, vascular endothelial growth factor; VEGFR, vascular endothelial growth factor receptor.

Fortunately, a study showed that the combination of anti-PD-1 drugs and anti-VEGFR-2 drugs promotes vascular normalization and enhances antitumor immune responses in HCC (62). In orthotopic HCC models in mice, Kohei Shigeta et al. revealed that combination therapy (anti-PD-1 antibody and anti-VEGFR-2 antibody) can significantly improve survival (HR = 0.054, 95% CI: 0.006–0.46, *p* = 0.007) compared with either treatment alone. Further research found that this combination regimen can reprogram the immune microenvironment by increasing CTLs function and CTLs/Tregs ratio and changing the M1/M2 ratio of tumor-infiltrating macrophages (**Figure 2**). Interestingly, VEGFR-2 blockade predominantly upregulated PD-L1 expression in HCC cells and increased the PD-1 expression in tumor-infiltrating CD4^+^ T cells *via* interferon-gamma of the endothelial cells paracrine. Moreover, they also found that, when combined with anti-VEGFR-2 antibodies, the anti-PD-L1 antibodies can promote normalized vessel formation mediated by CD4^+^ T cells, which decreases hypoxia in HCC (62) (**Figure 2**). Therefore, immunotherapy in combination with the anti-VEGFR-2 drugs, such as the drugs mentioned above (sorafenib, lenvatinib, regorafenib, cabozantinib, sunitinib, linifanib, and brivanib), is another new promising direction in the treatment of HCC.

In an open-label, multicenter, single group assignment phase 1b study (KEYNOTE-524 trial, NCT 03006926, **Table 2**), the lenvatinib plus pembrolizumab combination showed promising antitumor activity [based on RECIST 1.1, ORR: 36.0% (95% CI, 26.6% to 46.2%); median DOR: 12.6 months (95% CI, 6.9 months to NE); median PFS: 8.6 months: median OS: 22.0 months] with an acceptable safety profile (grade ≥ 3 TRAEs occurred in 67.0% of patients) in patients with unresectable HCC (63, 64). Based on the encouraging results, the effect of the combination (pembrolizumab + lenvatinib) *vs*. lenvatinib as a first-line treatment for advanced HCC is under evaluation in an ongoing phase III LEAP-002 trial (NCT03713593, **Table 3**). In addition, a phase I study (NCT 02942329, **Table 2**) to assess the efficacy and safety of combination of apatinib (a VEGFR-2 inhibitor) and SHR-1210 (an anti-PD-1 monoclonal antibody) in participants with advanced HCC showed encouraging clinical activity (PR rate 54.5%; ORR 50.0%; DCR 85.7%) (65). To further confirm the efficacy of SHR-1210 combined with apatinib, an open-label, non-randomized phase II study (NCT03463876, **Table 3**) and a randomized, open-label, international, multicenter, phase III study (NCT03764293, **Table 3**) are ongoing to both evaluate the same combination of SHR-1210 and apatinib. At present, there are several ongoing trials to explore immunotherapy in combination with the VEGFR inhibitors, including nivolumab + sorafenib (NCT03439891), pembrolizumab + sorafenib (NCT03211416), nivolumab + lenvatinib (NCT03418922 and NCT03841201), pembrolizumab + regorafenib (NCT03347292), nivolumab + cabozantinib (NCT01658878), and atezolizumab + cabozantinib (NCT03755791) (**Table 3**).

**Table 2:** Completed clinical trials of combining immune checkpoint inhibitors with antiangiogenic drugs in HCC.

Treatment arms (*n*)	Phase		Disease Condition	Results	TRAEs	ClinicalTrials.gov Identifier
lenvatinib + Pem (104)		Ib	No prior systemic therapy for advanced/unresectable HCC	ORR: 36.7%; Median DOR: 12.6 mo; Median PFS: 8.6 mo; Median OS: 22 mo	Grade ≥ 3: 67%	NCT03006926
apatinib + SHR-1210 (42)		I	Advanced HCC	PR rate 54.5%; ORR 50.0%; DCR 85.7%	Grade ≥ 3: 58%	NCT02942329
Arm A: Ate + Bev (104) Arm F: Ate +Bev (60) *vs*. Ate (59)		Ib	Advanced HCC who has received no prior systemic treatment	Arm A: ORR 36% Arm F: PFS 5.6 *vs*. 3.4 mo [HR 0.55, 80% CI, 0.40–0.74, *p* = 0.0108]	Arm A: Any-grade: 68%; Grade 3–4: 20%; Grade 5: 3% Arm F: Any-grade: 41%; Grade 3–4: 5%; Grade 5: 0%	NCT02715531
Ate+Bevb (336) *vs*. sorafenib (165)		III	Untreated locally advanced or metastatic HCC	PFS: 6.8 *vs*. 4.3 mo [HR 0.59; 95% CI, 0.47 to 0.76; *p* < 0.001]; OS at 12 mo: 67.2% (95% CI, 61.3 to 73.1) *vs*. 54.6% (95% CI, 45.2 to 64.0)	Grade 3–4: 56.5% *vs*. 55.1%; Grade 5: 4.6% *vs*. 5.8%	NCT03434379

Ate, atezolizumab; Bev, bevacizumab; CI, confidence interval; DCR, disease control rate; HCC, hepatocellular carcinoma; HR, hazard ratio; mo, months; ORR, objective response rate; Pem, pembrolizumab; PFS, progression-free survival; PR, partial response; TRAEs, treatment-related adverse events; OS, overall survival.

**Table 3:** Ongoing studies incorporating ICIs and antiangiogenic agents in HCC.

Drugs	Phase	Patients (*n*)	Study design	Endpoint		ClinicalTrials.gov Identifier
ICIs+Anti-VEGFR therapy						
Sorafenib Nivolumab	II	Advanced HCC (12)	Open-label, non-randomized, multicenter, sequential assignment: sorafenib + nivolumab as first line of systemic therapy	Primary: MTD; ORR Secondary: DCR; AEs; OS; PFS	NCT03439891	
Sorafenib Pembrolizumab	Ib/II	Advanced HCC (27)	Open-label, single group assignment: sorafenib + pembrolizumab	Primary: ORR Secondary: OS; TTP	NCT03211416	
Lenvatinib Nivolumab	Ib	Advanced HCC (30)	Open-label, non-randomized, multicenter, sequential assignment: lenvatinib + nivolumab	Primary: Safety Secondary: ORR	NCT03418922	
Lenvatinib Nivolumab	II	Advanced HCC (50)	Open-label, single-arm, multicenter, single group assignment: lenvatinib + nivolumab	Primary: ORR (RECIST 1.1); safety Secondary: ORR (iRECIST); TTP; PFS; OS	NCT03841201	
Lenvatinib Pembrolizumab	III	Advanced HCC (750)	Multicenter, randomized, double-blinded, parallel assignment: Lenvatinib + pembrolizumab *vs*. lenvatinib + placebo	Primary: PFS; OS Secondary: ORR; DOR; DCR; AEs	NCT03713593	
Regorafenib Pembrolizumab	Ib	Advanced HCC with no prior systemic therapy (57)	Open-label, non-randomized, multicenter, sequential assignment: regorafenib +pembrolizumab	Primary: AEs; Secondary: MTD; PFS; TTP; OS; ORR; DCR; DOR	NCT03347292	
Cabozantinib Sorafenib Nivolumab Ipilimumab	I/II	Advanced HCC (1097)	Open-label, non-randomized, multicenter, non-comparative, sequential assignment: nivolumab; sorafenib; cabozantinib+nivolumab; cabozantinib + nivolumab + ipilimumab	Primary: AEs; ORR Secondary: CR; DOR; DCR; TTR; TTP; PFS; OS	NCT01658878	
Cabozantinib Atezolizumab Sorafenib	III	Advanced HCC who has not received previous systemic anticancer therapy (740)	Open-label, randomized, controlled, parallel assignment: cabozantinib + atezolizumab *vs*. sorafenib; cabozantinib *vs*. sorafenib	Primary: PFS; OS (cabozantinib + atezolizumab *vs*. sorafenib) Secondary: PFS (cabozantinib *vs*. sorafenib)	NCT03755791	
Apatinib Camrelizumab	II	Advanced HCC (190)	Open-label, multicenter, single group assignment: apatinib + camrelizumab as second-line treatment	Primary: ORR Secondary: DOR; DCR; OS; PFS	NCT03463876	
Apatinib Sorafenib Camrelizumab	III	Advanced HCC who has not previously received systemic therapy (510)	Open-label, randomized, international, multicenter, parallel assignment: camrelizumab + apatinib *vs*. sorafenib as first-line therapy	Primary: OS; PFS Secondary: TTP; ORR; DCR; DOR; AEs	NCT03764293	
ICIs+Anti-VEGF therapy						
Bevacizumab Atezolizumab	III	HCC at high risk of recurrence after surgical resection or ablation (662)	Open-label, multicenter, randomized, parallel assignment: atezolizumab + bevacizumab *vs*. active surveillance as adjuvant therapy	Primary: RFS Secondary: OS; TTR	NCT04102098	
Bevacizumab Durvalumab	III	HCC who are at high risk of recurrence after curative hepatic resection or ablation (888)	Randomized, double-blind, placebo-controlled, multicenter, parallel assignment: durvalumab + bevacizumab *vs*. durvalumab *vs*. placebo as adjuvant therapy	Primary: RFS (durvalumab *vs*. placebo) Secondary: RFS (durvalumab + bevacizumab *vs*. placebo) OS; TTR	NCT03847428	
IBI305 (anti-VEGF antibody) Sorafenib Sintilimab (anti-PD-1 antibody)	II/III	Advanced HCC (595)	Open-label, multicenter, randomized, parallel assignment: sintilimab + IBI305 *vs*. sorafenib	Primary: OS; PFS Secondary: ORR; DCR; DOR;	NCT03794440	

AEs, adverse events; DCR, disease control rate; DOR, duration of response; HCC, hepatocellular carcinoma; ICIs, immune checkpoint inhibitors; iRECIST, modified RECIST1.1 for immune-based therapeutics; MTD, maximum tolerated dose; ORR, objective response rate; OS, overall survival; PFS, progression-free survival; RECIST, response evaluation criteria in solid tumors; RFS, recurrence-free survival; TTP, time to tumor progression; TTR, time to recurrence.

Another type of antiangiogenic drug is the anti‐VEGF monoclonal antibody, such as bevacizumab. A study found that the combination with ICIs and anti‐VEGF monoclonal antibody presents a potential synergistic antitumor effect by targeting the tumor immune microenvironment (66, 67). On the one hand, VEGF blockade not only can enhance antigen presentation by promoting differentiation and maturation of DCs, but also activate cytotoxic CD8^+^ T cells (68, 69). Moreover, the VEGF inhibitors promote lymphocyte infiltration into tumors through restoration of microvessel density and regulation of some endothelial adhesion molecules in tumor vessels, thus enhancing the efficacy of immunotherapy (70, 71). In addition, antiangiogenic agents can reduce immunosuppression *via* the following mechanisms: restriction of Tregs chemotaxis and accumulation into tumors by suppressing the expression of IL-1β, IL-6, and CXCL1 (72), and alleviation of hypoxia through the normalization of the tumor vasculature relieving the immunosuppression exerted by TAMs, MDSCs, and Tregs (73) (**Figure 2**). On the other hand, ICIs can facilitate tumor vessel normalization, which can be reflected in the reduction of tumor vascular density, improvement of vessel perfusion, as well as alleviation of tumor tissue hypoxia, through the activation of CD4^+^ T cells and the activation of IFN-γ signaling pathway due to CD8+ effector T cells (73, 74). A study reveals that, when anti-VEGF agents are used to treat cancer, the hypoxia due to excessive vessel regression will cause some cells (MDSCs, M2-TAMs, as well as Tregs) associated with immunosuppression to stimulate angiogenesis *via* upregulating the expression of pro-angiogenic factors, which results in resistance to anti-VEGF therapy (75) (**Figure 2**). Then, the addition of immunotherapy to anti-VEGF drugs can reverse resistance to anti-VEGF agents by inhibiting these inhibitory cells and alleviating immunosuppression. Therefore, given the synergistic interactions between the two treatments, the combination regimen (immunotherapy + anti-VEGF therapy) is expected to be a promising treatment for patients with HCC.

Bevacizumab (an anti-VEGF monoclonal antibody) mainly suppresses tumor angiogenesis by binding to VEGF and interfering with the interaction between VEGF and VEGFR on the surface of endothelial cells. In an open-label, multicenter phase Ib trial (GO30140 study, NCT 02715531, **Table 2**) involving atezolizumab combining with bevacizumab, arms A and F enrolled the patients with advanced HCC who have received no prior systemic treatment (76). For arm A (bevacizumab + atezolizumab every 3 weeks) with 104 patients, primary endpoint ORR was 36% (37 patients) with 76% of responses still underway. Any-grade TRAEs were seen in 91 (88%) patients; 41 (39%) patients had grade 3–4 TRAEs. Grade 5 TRAEs occurred in three (3%) patients. In arm F, patients were randomized 1:1 to atezolizumab (1,200 mg IV q3w) plus bevacizumab (15 mg/kg IV q3w) or atezolizumab (1,200 mg IV q3w) monotherapy, until unacceptable toxicity or progression of disease. Arm F showed that the combination group significantly improved the primary endpoint median PFS (5.6 *vs*. 3.4 months, HR 0.55, 80% CI, 0.40–0.74, *p* = 0.0108) compared with atezolizumab. Combining atezolizumab with bevacizumab has a manageable safety profile: in the combination group and monotherapy, the incidence of any-grade TRAEs is 68% and 41%, respectively; grade 3–4 TRAEs were seen in 12 (20%) patients and 3 (5%) patients, respectively. There was no grade 5 TRAEs in arm F (76).

Recently, there is a crucial trial (IMbrave150) that shows that the treatment with bevacizumab and atezolizumab leads to better PFS and OS than single-agent sorafenib in patients with locally advanced or metastatic HCC who have received no prior systemic treatment (11, 77). In this global, open-label, phase III trial (NCT 03434379, **Table 2**), patients were randomly assigned in a 2:1 ratio to the atezolizumab plus bevacizumab group (*n* = 336) and sorafenib group (*n* = 165) until unacceptable toxicity or loss of clinical benefit. This study revealed that the combination of atezolizumab and bevacizumab achieved a better median PFS: 6.8 *vs*. 4.3 months (HR 0.59; 95% CI, 0.47 to 0.76; *p* < 0.001) compared with sorafenib alone. Of note, the OS at 12 months was higher for atezolizumab plus bevacizumab compared with sorafenib (67.2% *vs*. 54.6%). Moreover, the ORR was 27% *vs*. 12% (*p* < 0.0001) under independent assessment with RECIST 1.1 and 33% *vs*. 13% (*p* < 0.0001) under HCC-specific mRECIST for atezolizumab + bevacizumab *vs*. sorafenib, respectively (78). The DCR is 73.6% and 55.3%, respectively. Notably, they found that atezolizumab combined with bevacizumab can significantly delay deterioration of physical functioning: median time to deterioration (TTD) is 13.1 *vs*. 4.9 months (HR, 0.53; 95% CI, 0.39 to 0.73), quality of life: median TTD is 11.2 *vs*. 3.6 months (HR, 0.63; 95% CI, 0.46 to 0.85), and role functioning: median TTD, 9.1 *vs*. 3.6 months (HR, 0.62; 95% CI, 0.46 to 0.84) compared with sorafenib (79). As for safety, grade 3–4 TRAEs were seen in 329 (56.5%) patients treated with atezolizumab + bevacizumab and in 156 (55.1%) patients who received sorafenib, and other high-grade side effects were infrequent (77).

Due to the exciting findings from these studies, the FDA approved the treatment regimen with atezolizumab and bevacizumab as an updated first-line systemic treatment for the treatment of advanced HCC. In the context of combining ICIs with bevacizumab, further phase III trials are currently ongoing. In particular, the IMbrave050 trial (NCT04102098, **Table 3**), an open-label, randomized, multicenter, phase III study, is currently ongoing to evaluate the efficacy and safety of adjuvant therapy with bevacizumab and atezolizumab compared with active surveillance in high risk of recurrence HCC patients after curative treatment (surgical resection or ablation), while EMERALD-2 trial (NCT03847428, **Table 3**), a randomized, double-blind, placebo-controlled, multicenter, phase III study, is assessing the efficacy and safety of durvalumab alone or combined with bevacizumab in the same adjuvant setting. Finally, a randomized, open-label, multicenter study (ORIENT-32 trial, NCT03794440) is testing the combination of IBI305 (anti-VEGF monoclonal antibody) with sintilimab (anti-PD-1 monoclonal antibody) in participants with advanced HCC as the first-line treatment compared with sorafenib (**Table 3**).

## Combining With Locoregional Therapies

Locoregional therapies, such as radiofrequency ablation (RFA) (80), transarterial chemoembolization (TACE) (81), stereotactic body radiotherapy (SBRT) (82), and selective internal radiation therapy (SIRT) (83), play a key role in the treatment of patients with HCC. These locoregional treatments not only can destroy primary tumors but also facilitate antitumor immunity by releasing the neoplasm antigens from killed tumor cells (84). Interestingly, ionizing radiation exerts an abscopal effect in several tumors, such as lung adenocarcinoma (85), which refers to the rejection and regression of unirradiated metastatic lesions after the irradiation of a distant tumor location (86, 87). More importantly, the addition of immunotherapy to radiotherapy can boost the abscopal effect (88) and, in turn, radiotherapy can enhance the effect of immunotherapy (89). For the above reasons, it is believed that the combination of locoregional therapies with immunotherapeutic agents can act synergistically.

In a phase I/II trial, the safety and effectiveness of tremelimumab combined with RFA or TACE for HCC were examined (90) (NCT01853618, **Table 4**). Thirty-two enrolled patients received tremelimumab [at two dose levels (3.5 and 10 mg/kg) every 4 weeks for six cycles, followed by infusions every 3 months] and an ablation (on day 36, subtotal chemoablation or radiofrequency ablation). Data from this study demonstrated that 26.3% (5/19) patients reached a firm PR. For the refractory HCC population, 6- and 7-month probabilities of tumor PFS were 57.1 and 33.1%, respectively, with a median TTP of 7.4 months (95% CI: 4.7 months to 19.4 months) and a median OS of 12.3 months (95% CI: 9.3 months to 15.4 months). Interestingly, 12 of 14 patients with quantifiable HCV experienced an obvious reduction in viral load and 6-week tumor biopsies showed a marked increase in CD8^+^ T cells in patients who presented a clinical benefit alone. Moreover, for the patients with a clinical benefit, a clear accumulation of intratumoral CD8^+^ T cells was found in 6-week tumor biopsies (90). In addition, another phase II study revealed that additional ablation could enhance the effect of immunotherapy in HCC (NCT03939975, **Table 4**) (91). Of all 50 patients who received an anti-PD-1 inhibitor (pembrolizumab/nivolumab) as second-line treatment, 33 cases with SD or atypical response to anti-PD-1 drugs were treated with subtotal thermal ablation. The results of this study are encouraging. Additional ablation improved efficacy with a higher response from 10% to 24% (12/50) and tolerable toxicity. The media PFS, OS, and TTP were 5.0 months (95% CI, 2.9–7.1), 16.9 months (95% CI, 7.7–26.1), and 6.1 months (95% CI, 2.6–11.2), respectively (91).

**Table 4:** Completed clinical trials of combining immune checkpoint inhibitors with locoregional therapies in HCC.

Treatment arms (*n*)	Phase		Disease Condition	Results	ClinicalTrials.gov Identifier
Tre + RFA or TACE (32)		I/II	Advanced HCC	PR: 26.3%; 6 months PFS: 57.1%; MTP: 7.4 months (95% CI: 4.7–19.4); median OS: 12.3 months (95% CI: 9.3–15.4)	NCT01853618
Pem or Niv + thermal ablation (50)		II	Advanced HCC	ORR:10% *vs*. 24% (Pem or Niv *vs*. Pem or Niv + thermal ablation); PFS: 5 months (95% CI, 2.9–7.1); MTP: 6.1 months (95% CI, 2.6–11.2); OS: 16.9 months (95% CI, 7.7–26.1)	NCT03939975
Nivo + Y90-radioembolization (40)		II	Asian patients with advanced HCC	ORR: 31%; DCR: 58.3%; median PFS: 4.6 months (95% CI 2.3–4.8); median OS: 15.1 months (95% CI 7.8–NE); 3/4 grade TRAEs: 11%	NCT03033446

CI, confidence interval; DCR, disease control rate; HCC, hepatocellular carcinoma; MTP, median time to tumor progression; Niv, nivolumab; ORR, objective response rate; Pem, pembrolizumab; PFS, progression-free survival; PR, partial response; RFA, radiofrequency ablation; TACE, transarterial chemoembolization; TRAEs, treatment-related adverse events; Tre, tremelimumab; OS, overall survival.

At present, TACE still serves as the standard therapeutic regimen for intermediate-stage HCC (92). Based on the reasons mentioned before, it is encouraging that combining ICIs with TACE can improve the effect of the standard treatment for HCC. In a phase I/II PETAL clinical trial, the combination of conventional TACE followed by pembrolizumab had a tolerable safety profile without cumulative side effects (93). The study of TACE combined with immunotherapy is conducted in several trials. The phase II trial IMMUTACE study evaluates the safety and efficacy of the nivolumab combined with TACE in patients with intermediate-stage HCC as first-line treatment (NCT03572582, **Table 5**). Moreover, both phase II trials, including combined ICIs (durvalumab + tremelimumab) with ablative therapies (TACE, RFA, or cryoablation) in participants with HCC or biliary tract cancer (NCT02821754) and pembrolizumab plus local ablation [RFA, microwave ablation (MWA), brachytherapy, or TACE] in patients with early-stage HCC (NCT03753659), are underway. Additionally, there are two ongoing clinical studies evaluating the effect of nivolumab + TACE (NCT03143270) and tremelimumab + durvalumab following TACE (NCT03638141) in patients with advanced HCC (**Table 5**).

**Table 5:** Ongoing clinical trials combining immunotherapy and locoregional therapy in HCC.

Treatment	Phase	Setting (*n*)	Endpoint	ClinicalTrials.gov Identifier
Nivolumab + TACE	I	Advanced HCC (14)	Primary: safety and feasibility	NCT03143270
Nivolumab + TACE	II	Intermediate stage HCC (49)	Primary: ORR Secondary: PFS; TTP; OS	NCT03572582
Durvalumab+tremelimumab following TACE	II	Advanced HCC (30)	Primary: ORR Secondary: PFS; PR; CR; OS; safety	NCT03638141
Durvalumab+tremelimumab+ ablative therapies (TACE, RFA or cryoablation)	II	HCC or BTC (90)	Primary: PFS Secondary: safety	NCT02821754
Pembrolizumab+local ablation (RFA, MWA, brachytherapy or TACE)	II	Early-stage HCC (30)	Primary: ORR Secondary: TTR; Recurrence free survival; OS; AEs	NCT03753659
Pembrolizumab+ SIRT (Y-90 radioembolization)	I	HCC with poor prognosis not eligible for liver transplant or surgical resection with well compensated liver function (30)	Primary: PFS Secondary: safety; TTP; ORR; OS	NCT03099564
Nivolumab+ Y-90 Radioembolization	I	Advanced HCC (27)	Primary: MTD; ORR Secondary: AEs; PFS; DCR	NCT02837029
Nivolumab+ Y-90 Radioembolization	II	Asian patients with advanced HCC (40)	Primary: RR Secondary: TTR; DOR; TTP; PFS; OS; AEs	NCT03033446
Nivolumab after SIRT using SIR-spheres	II	Unresectable HCC (41)	Primary: AEs Secondary: RR; DCR; DOR; TTP; PFS; OS	NCT03380130
Pembrolizumab+SBRT	II	Advanced HCC (30)	Primary: ORR Secondary: RR; PFS; OS	NCT03316872
SBRT followed by sintilimab *vs*. SBRT	II/III	HCC with portal vein invasion after arterially directed therapy (116)	Primary: 24-week PFS rate Secondary: PFS; OS; ORR; DCR; DOR	NCT04167293
TACE+apatinib+camrelizumab	II	C-staged HCC in BCLC classification (84)	Primary: ORR Secondary: DOR; DCR; PFS; OS; AEs	NCT04191889
Lenvatinib+pembrolizumab +TACE *vs*. TACE	III	Incurable/non-metastatic HCC (950)	Primary: PFS; OS Secondary: ORR; DCR; DOR; TTP; AEs	NCT04246177
Arm A: TACE + durvalumab; Arm B: TACE + durvalumab + bevacizumab; Arm C: TACE	III	Locoregional HCC (710)	Primary: PFS (Arm B *vs*. Arm C) Secondary: PFS (Arm A *vs*. Arm C); OS	NCT03778957

AEs, adverse events; BTC, biliary tract carcinomas; CR, complete response; DCR, disease control rate; DOR, duration of response; HCC, hepatocellular carcinoma; MWA, microwave ablation; MTD, maximum tolerated dose; ORR, objective response rate; OS, overall survival; PFS, progression-free survival; PR, partial response; RFA, radiofrequency ablation; RFS, recurrence-free survival; RR, response rate; SBRT, stereotactic body radiotherapy; SIRT, selective internal radiation therapy; TTP, time to tumor progression; TACE, transarterial chemoembolization; TTR, time to recurrence; Y-90, Yttrium-90.

Radiotherapy, mainly including SIRT and SBRT, is another local treatment approach used in patients with HCC. Recently, a CA 209-678 study, the open-label, single-center, nonrandomized phase II trial, is carried out to explore the effect of Y90-radioembolization combined with nivolumab in Asian patients with advanced HCC (NCT03033446, **Table 4**) (94). The results from this study revealed the encouraging clinical activity [ORR of 31%, DCR of 58.3%, median PFS of 4.6 months (95% CI 2.3–4.8 months), and median OS of 15.1 months (95% CI 7.8–NE)], with safety profile (only 11% had grade 3/4 TRAEs) (94). With these exciting results, several trials, such as SIRT in combination with pembrolizumab (NCT03099564) and SIRT combined with nivolumab (NCT02837029 and NCT03033446), are currently underway (**Table 5**). Another study (NCT03380130), a phase II clinical trial whose purpose is to investigate the efficacy of nivolumab following SIRT for the treatment of patients with unresectable HCC, had completed the enrollment. As for the combination of SBRT with ICIs, there are two ongoing clinical trials, including the phase II study (NCT03316872) that assesses the effect of the pembrolizumab combined with SBRT in subjects with advanced HCC who have experienced disease progression after treatment with sorafenib and the phase II/III study (NCT04167293) to test the efficacy of SBRT followed by anti-PD1 antibody for HCC (**Table 5**).

It is noteworthy that not all effects of locoregional therapies favor antitumor immunity because local therapies can cause increases in hypoxia and vascular permeability and the release of some cytokines (VEGFs and TGFβ), which can inhibit the efficacy of the immune response against tumor (86). Therefore, the combination of anti-VEGF/VEGFR agents with locoregional therapy and immunotherapy is expected to be a promising option for HCC. Currently, a phase II trial is testing the efficacy and safety of TACE plus camrelizumab and apatinib for C-staged HCC in BCLC classification (NCT04191889). In addition, the LEAP-012 study is a randomized, multicenter, double-blinded, phase III trial to evaluate the safety and effectiveness of TACE with lenvatinib and pembrolizumab *vs*. TACE alone in patients with non-metastatic/incurable HCC (NCT04246177). Another multicenter, randomized, double-blind, phase III study of TACE with bevacizumab and durvalumab in patients with locoregional HCC is in the process (NCT03778957) (**Table 4**).

## Conclusion

More than 60% of patients with HCC are diagnosed in advanced stages due to the absence of obvious symptoms in the early stages of HCC (95), resulting in an extremely low 5-year OS rate (less than 16%) (96). Although the emergence of some small-molecule drugs, such as sorafenib, lenvatinib, and other TKI, has improved the treatment of HCC, the survival benefit is only 3 months due to drug resistance. Hence, it is urgent to find a therapeutic strategy to improve the efficacy and prognosis of HCC. Fortunately, immunotherapy has demonstrated significant potential in the treatment of HCC in recent years. However, immunotherapy alone presents low response. Thereby, multiple ICIs and ICIs combined with other therapies, such as antiangiogenic drugs (sorafenib, lenvatinib, regorafenib, cabozantinib, sunitinib, linifanib, brivanib, tivantinib, and ramucirumab, and bevacizumab) and locoregional therapies (RFA, TACE, SBRT, and SIRT), represent a novel modality for the treatment of HCC, which may achieve greater efficacy through multiple synergistic mechanisms. Based on the differences in timing, location, and nonoverlapping effects of the two immune checkpoint pathways, including PD-1/PD-L1 and CTLA-4 immune checkpoints, combining different ICIs may act synergistically in the treatment of HCC. Moreover, exciting results are reported for ipilimumab in combination with nivolumab as a second-line treatment; thereby, this combination regime has been approved by the FDA. Regarding ICIs in combination with antiangiogenic drugs, compelling evidence supports such combinations as the treatment for HCC due to synergistic modulation of both the vasculature and the immune microenvironment of tumor. Furthermore, the addition of ICIs to locoregional treatments may represent a useful strategy in HCC. On the one hand, locoregional treatments can reinforce antitumor immunity by releasing neoplasm antigens from killing tumor cells. On the other hand, ICIs can enhance the antitumor immune response induced by locoregional treatments. For these combinations mentioned above, a main problem needing to be resolved is how to optimize the dose and schedule of the combination therapy in the treatment of patients with HCC. Currently, many ongoing trials are investigating whether these combinations with ICIs will improve the survival of patients with HCC, and we will await the encouraging outcomes and the realization of clinical application to benefit patients.

Finally, it is worth noting that although immunotherapy has improved the response rate in the treatment of HCC, the majority of patients still fail to get benefit from this promising treatment (97). Therefore, there is an urgent need for effective predictive biomarkers to identify patients likely to benefit from this therapy in the next few years. To our knowledge, PD-L1 expression is widely used today for the selection of anti-PD-1 therapy in patients with non-small cell lung cancer (98). As for HCC, several studies have demonstrated that PD-L1 expression is associated with response to PD-1 inhibitor therapy (45, 46, 48). Andrew X Zhu et al. found that, through the use of CPS with tumor cell scoring and immune cell (TILs and macrophage) scoring, the predictive value of the PD-L1 immunohistochemistry assay could improve, which needs to be validated in larger studies (48). In addition, a study revealed a significant correlation between tumor mutation burden (TMB) and clinical outcomes after PD-1 inhibitors (99). Thus, TMB will probably serve as another potential biomarker in response to ICIs in the treatment of HCC. Besides PD-L1 expression and TMB, Daniela Sia et al. found that a subgroup of HCCs with markers of the inflammatory response, such as fewer chromosomal aberrations, and markers of cytolytic activity, might be susceptible to immunotherapy (100). We look forward to the discovery of effective predictive biomarkers to identify those patients with HCC that really benefit from ICIs to receive this therapy in the near future.

## Author Contributions

All authors contributed to the article and approved the submitted version.

## Funding

This work was supported by the Excellent Young Teachers Program of China Medical University (No. QGZ2018060).

## Conflict of Interest

The authors declare that the research was conducted in the absence of any commercial or financial relationships that could be construed as a potential conflict of interest.

## Publisher’s Note

All claims expressed in this article are solely those of the authors and do not necessarily represent those of their affiliated organizations, or those of the publisher, the editors and the reviewers. Any product that may be evaluated in this article, or claim that may be made by its manufacturer, is not guaranteed or endorsed by the publisher.
